# Athena’s Pages on Neonatal Appendicitis

**Published:** 2015-04-01

**Authors:** G Raghavendra Prasad

**Affiliations:** Department of Pediatric Surgery, Deccan College of Medical Sciences and Princess Esra Hospital, Hyderabad, India

**Dear Sir**

I keenly read the Athena's page article on neonatal appendicitis (NA) [1]. While appreciating Athena's collective review on neonatal appendicitis, I have the following reservations:

Athena has mixed apples and oranges in her article on NA. She has not given definite conclusions, rather left many loose ends at the end, making with unsupported statements. NA is rare. I feel it is probably Systemic Inflammatory Response Syndrome (SIRS) initiated in gut by common enemies of neonate, namely, hypoxia, hypothermia, acidosis and sepsis. This is supported by the fact that occult coagulopathy is seen in many of these cases. I have seen only 3 cases of NA in his 35 years of practice. Even neonatal necrotizing enterocolitis (NEC) is not a pure intestinal disease; it is an intestinal manifestation of endothelial activation, platelet and neutrophil, macrocyte activation initiated by intestinal and systemic factors. I felt that Athena concentrated more on numbers rather than the underlying pathophysiology. All the signs described by Athena for early diagnosis of NA are common to all sources with intra-abdominal pus; I will be happy if Athena responds to this. I strongly feel that budding pediatric surgeons need to know the pathogenesis of neonatal appendicitis. I wish Athena could have added some comments on the currently accepted pathogenesis of neonatal acute appendicitis.

## Footnotes

**Source of Support:** Nil

**Conflict of Interest:** None

